# Introducing an Integrated Model of Adults’ Wearable Activity Tracker Use and Obesity Information–Seeking Behaviors From a National Quota Sample Survey

**DOI:** 10.2196/23237

**Published:** 2021-09-29

**Authors:** Bokyung Kim, Seoyeon Hong, Sungwook Kim

**Affiliations:** 1 Department of Public Relations & Advertising Ric Edelman College of Communication & Creative Arts Rowan University Glassboro, NJ United States; 2 Department of Math, Physics, and Statistics Misher College of Arts and Sciences University of the Sciences in Philadelphia Philadelphia, PA United States

**Keywords:** wearable activity tracker, wearable health technology, obesity, health belief, health belief model, Technology Acceptance Model, online information seeking

## Abstract

**Background:**

Research from multiple perspectives to investigate adults’ use of wearable activity-tracking devices is limited. We offer a multiperspective model and provide empirical evidence of what leads to frequent usage of wearable health technologies from a large, nationally representative survey sample.

**Objective:**

This study aims to explore factors affecting the use of wearable activity-tracking devices among health consumers from the perspectives of individual health beliefs (perceived severity, perceived susceptibility, perceived benefits, and self-efficacy) and information-seeking behaviors.

**Methods:**

Our Integrated Model of Wearable Activity Tracker (IMWAT) use and proposed hypotheses were validated and tested with data collected from a telephone survey with a national quota sample. The data were analyzed using a variety of statistical techniques, including structural equation analysis.

**Results:**

The sample comprised 2006 participants. Our results showed that the perceived benefits of physical activity, perceived susceptibility, and self-efficacy toward obesity were significant predictors of information-seeking behaviors, which, in turn, mediated their effects on the use of wearable activity trackers. Perceptions of obesity severity directly promoted wearable device usage.

**Conclusions:**

This study provided a new and powerful theoretical model that combined the health beliefs and information-seeking behaviors behind the use of wearable activity trackers in the adult population. The findings provide meaningful implications for developers and designers of wearable health technology products and will assist health informatics practitioners and obesity prevention communicators.

## Introduction

### Background

Obesity is regarded as an ongoing international health problem. Numerous studies have explored behavioral determinants of obesity such as an individual’s psychological beliefs, unhealthy dietary habits, stress levels, and inadequate physical activity [1]. Physical inactivity is a major contributing factor to the rising health care costs of obesity and significant increase in overweight in the adult population [2]. Such inactivity and low cardiorespiratory fitness can cause subsequent chronic diseases such as type 2 diabetes, coronary heart disease, and stroke [3]. Obesity requires constant care to manage the serious health risks associated with the symptom, yet obese adults are generally the persons primarily responsible for modifying their own lifestyle and self-managing aggressive interventions [4,5].

Scientists introduced the concept of wearable health technology by suggesting that this type of technology can provide a meaningful solution to obesity issues [6,7]. Wearable health technology refers to an electronic device or technology, incorporated into accessories, that can be directly worn on the body [8], mainly for self-tracking and self-monitoring purposes [9]. Yet, the scope of wearable health technology is very broad and encompasses many aspects of hardware and software, including mobile apps, wearable sensors, and devices.

Many wearable health devices have been developed to detect and promote physical movement. Such wearable technology delivers accurate physical activity data and changes in dietary intake compared to the conventional method of collecting health information [10,11]. Previous medical and informatics studies have mainly focused on the development and implementation of wearable fitness-tracking devices such as Fitbit [12-14]. Yet, theoretical research about the adoption and actual usage of wearable activity trackers is relatively sparse. Namely, much is still unknown about the multifactored mechanism that promotes use of wearable physical activity trackers among both obese adults and healthy consumers [15].

Thus, this study aims to fill this void; we begin by reviewing the prevailing consensus regarding psychological factors to predict obesity prevention behaviors from the Health Belief Model (HBM) [16,17] and connect the HBM with literature on information seeking. To this end, we propose an integrated model of wearable activity tracker use to describe how psychological beliefs influence people’s actions in seeking obesity-related health information online, which, in turn, leads to their use of wearable fitness trackers.

### Psychological Factors Pertaining to Wearable Health Technology Use

To develop a theoretical model of health consumers’ wearable health device use, this paper adopts the HBM as a theoretical framework to understand the factors that trigger usage. The HBM was one of the first and best-known social cognition models to explain health-related behaviors [18]. This model was initially formulated in the 1950s to explain low participation in disease prevention programs by examining individual motivations toward behaviors that could improve health or prevent illness.

The HBM explains certain beliefs in regard to threats to oneself (personal threat), together with belief in the effectiveness of a proposed behavior, and predicts the likelihood of engaging in that behavior [19]. In doing so, the HBM provides a cognitive framework that views people as rational individuals who have multidimensional antecedents regarding whether to perform a healthy behavior or not.

Applying this model to the obesity context, perceived susceptibility refers to the degree to which individuals perceive themselves to be susceptible to being obese; perceived severity refers to perceptions on risks or diseases among those who are overweight; perceived barriers equate to strong barriers that prevent individuals from obtaining obesity treatment or practicing intervention behaviors; and perceived benefits are one’s understanding of the tangible benefits of health behavior change such as regular exercise to prevent obesity [16,17,20]. Rosenstock and his colleagues [21] later suggested self-efficacy, a separate independent variable along with the traditional health belief variables, and defined it as “the conviction that one can successfully execute the behavior required to produce the outcomes” [21].

The health belief dimensions can provide reliable, though weak or varying, predictions of health behaviors [20,22]. For example, a meta-analysis indicated that self-efficacy (*r*=0.21), perceived susceptibility (*r*=0.15), perceived benefits (*r*=0.13), and perceived severity (*r*=0.08) were found to be significant factors across previous literature [22]. Similarly, a meta-analysis of 18 studies revealed perceived severity (*r*=0.14), as well as perceived benefits (*r*=0.11) and barriers (*r*=0.22), to consistently be the strongest predictors of healthy behaviors, while perceived susceptibility was the weakest predictor [20].

### Linkage Between Health Beliefs, Health Information Seeking, and Behavior Change

Research on health information and behavior change has identified antecedents of individuals’ health information–seeking behaviors. Health information seeking is defined as the purposive acquisition of health information from selected sources for determining one’s own health behaviors [23,24].

Research on this stream assumes a positive link between psychological factors from the HBM and health information seeking. For example, Johnson and Meischke [23] introduced a comprehensive model of health information seeking that integrates motivational drivers and health belief factors. From an online survey with a stratified random sample of 1004 mothers, Lee and Kim [25] applied Johnson and Meischke’s [23] information-seeking model to the context of diverse sources of childhood vaccination information [25]. The study incorporated psychological factors, such as perceived severity and self-efficacy, as the driving forces for health information seeking.

Mou and colleagues [26] also explored consumer acceptance of online health information and empirically tested their integrated health belief and information-seeking model. Their model confirmed the predictive power of psychological variables on health behaviors: not only did susceptibility, benefits, and severity perceptions positively lead to behavioral intentions to utilize online health information services, self-efficacy also moderated the effect of perceived severity on health information–seeking behaviors [26].

Given the theoretical link between health beliefs and health information–seeking behaviors, it is necessary to ask whether this link is still valid in other contexts, namely, obesity-related information seeking. There is also a theoretical uncertainty associated with some HBM variables in predicting various health behaviors. For instance, perceived susceptibility was the weakest predictor of health behaviors [20], while it had a strong positive effect on online health information seeking in Mou et al’s work [26].

### Health Beliefs and Health Information Seeking to Predict Wearable Activity Tracker Use

Many studies exploring the multifactored associations between health beliefs and health information–seeking behaviors have mainly focused on the Technology Acceptance Model (TAM) [27], which is a theoretical framework that explains the adoption of new health technology [25,28]. As mentioned, wearable health trackers were designed to promote a person’s healthy behaviors, while relatively few studies have explored what would directly lead to the adoption and actual usage of wearable activity trackers [11,29,30]. According to the TAM, an individual’s acceptance of health technology is determined by his or her intentions to use that technology. Behavioral intentions to use the technology, which, in turn, is driven by one’s attitude toward using the technology, impacts his or her actual use. The TAM is a very parsimonious model that is too obvious to test the linkage between one’s intention to use and actual adoption of technology. Hence, researchers recommend a careful approach when applying the TAM to other contexts and call for additional research that explores the multiple factors associated with one’s acceptance of various technologies and devices [31-33].

One noteworthy survey study of 728 members of 3 internet health portals in South Korea [34] developed and verified an extended TAM for health care, and added antecedents and mediating variables from the HBM to enhance the model’s explanatory power. The results showed that perceived threat significantly affected health consumers’ attitudes and behavioral intentions, while self-efficacy had a strong indirect impact on attitude and behavioral intention through the mediator of perceived threat [34].

Health information seekers are defined as people who search for information on health topics [35]. For example, if individuals perceive themselves as obese, they will need information to manage the situation, while that information would simultaneously reassure healthy individuals [36]. Internet users may search for general health information; however, the need for online health information seeking is greater among individuals who perceive their health condition to be severe [37]. Concerning the impact of obesity-related health beliefs (ie, HBM factors) on online health information seeking, we propose the following hypotheses:

Hypothesis 1: Perceived susceptibility will influence health information–seeking behaviors.

Hypothesis 2: Perceived severity will influence health information–seeking behaviors.

Hypothesis 3: Perceived benefits will influence health information–seeking behaviors.

Hypothesis 4: Self-efficacy will influence health information–seeking behaviors.

**Figure 1:**
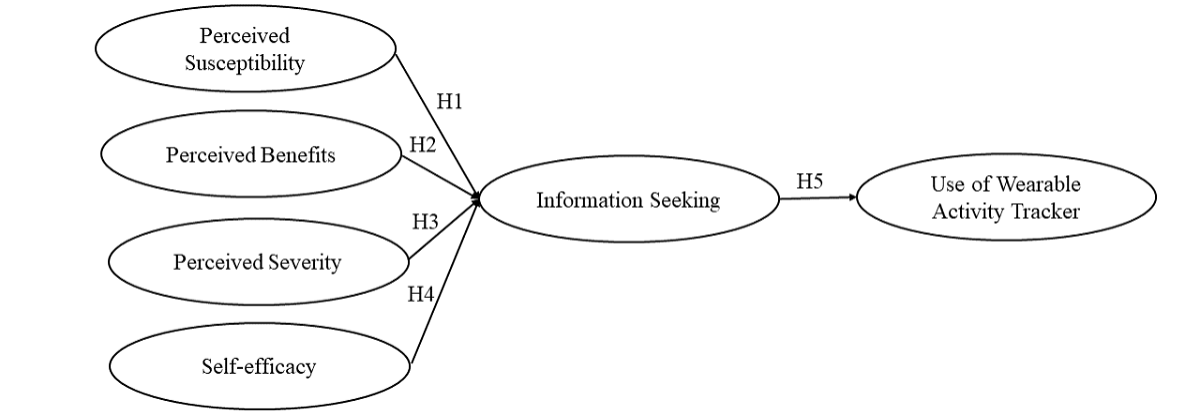
The proposed Integrated Model of Wearable Activity Tracker (IMWAT) use.

Then, we apply information seeking as a mediating variable between psychological factors (from the HBM) and prediction of wearable activity tracker usage in our theoretical model. Our integrated model considers an individual’s wearable health technology use as dependent on not only their psychological needs but health information–seeking behaviors [38]. Health information seekers are more likely to use wearable activity-tracking devices to monitor their food intake and physical activity levels if they perceive (1) themselves as susceptible to being obese, (2) the issue of obesity or overweight as severe, (3) benefits from such physical movements, and/or (4) any barriers that might hinder them from exercising regularly. Taken together, we present our fifth hypothesis as well as a research question (RQ) to test our proposed model (Figure 1):

Hypothesis 5: Obesity-related information seeking will influence individuals’ wearable activity tracker use.

RQ1: Is the Integrated Model of Wearable Activity Tracker (IMWAT) use an appropriate model to predict wearable technology use, mediated by information-seeking behaviors?

## Methods

### Data Collection

A telephone quota survey was conducted among adults who currently use wearable activity trackers (ie, respondents were asked the filtering question, “ Do you currently use a wearable health product such as Fitbit, Mi Band, or any sort of activity tracker?”), ensuring externally valid data. Participants were recruited by a reputable survey company, and researchers paid $130,000 for data collection, which was carried out over 2 months from August to September 2019. The survey company used random digit dialing to recruit participants; 50% of the data was retrieved from cell phones and the other 50% was collected from landline telephones. The average length of the survey was 24 minutes.

### Measurement

Perceived severity, perceived benefits, and perceived susceptibility were measured with a widely used set of 12 items from the HBM literature, answered on a 5-point Likert scale ranging from “strongly disagree” to “strongly agree” (Table 1) [39]. Self-efficacy was measured with an item asking participants to indicate their confidence in their ability to overcome or prevent obesity, using a 5-point scale ranging from 1=“not at all true for me“ to 5=“completely true for me” (Table 1) [40].

Health information seeking was measured with the 5-point scale ranging from 1=”not at all” to 5=”very frequently” (Table 1) [41]. Use of wearable activity trackers was measured with the filtering question, “Do you currently use a wearable health product such as Fitbit, Mi Band, or any sort of activity tracker?” [42].

**Table 1:** Measurement item.

Construct and item			Description		Reference
**Perceived susceptibility (PSU)**					Champion and Skinner [39]
	PSU-1	I have a somewhat high chance of having obesity.			
	PSU-2	I never worry about being obese.			
	PSU-3	It is fated that I will have obesity.			
	PSU-4	I can prevent myself from being obese.			
**Perceived severity (PSE)**					Champion and Skinner [39]
	PSE-1	I think obesity increases the risk of many health problems such as heart disease and diabetes.			
	PSE-2	I think obesity leads to suffering.			
	PSE-3	I think having obesity affects my family.			
	PSE-4	I think becoming obese affects my social life.			
	PSE-5	I think obesity in general is expensive to treat.			
**Perceived benefits (PBE)**					Champion and Skinner [39]
	PBE-1	I think screening all adults for obesity (such as through body mass index) detects obesity early.			
	PBE-2	I think regular exercise make a difference.			
	PBE-3	I think multicomponent behavioral obesity interventions do make a difference.			
**Self-efficacy (SE)**					Grace-Leitch and Shneyderman [40]
	SE-1	I have the ability to avoid obesity.			
	SE-2	I believe I can prevent an obesity condition.			
	SE-3	I am confident I will react in the right way if I have obesity.			
	SE-4	I have the ability to get and make sense of information about risks of being obese.			
**Information seeking (IS)**					Nikoloudakiet et al [41]
	IS-1	I seek obesity-related health information on the government department website such as the CDC^a^ or the NIH^b^.			
	IS-2	I seek obesity-related health information on social networking sites (eg, Facebook, Instagram, Twitter, etc).			
	IS-3	I seek obesity-related health information from online search engines such as Google.			

^a^CDC: Centers for Disease Control and Prevention.

^b^NIH: National Institutes of Health.

## Results

### Demographic Characteristics

**Figure 2:**
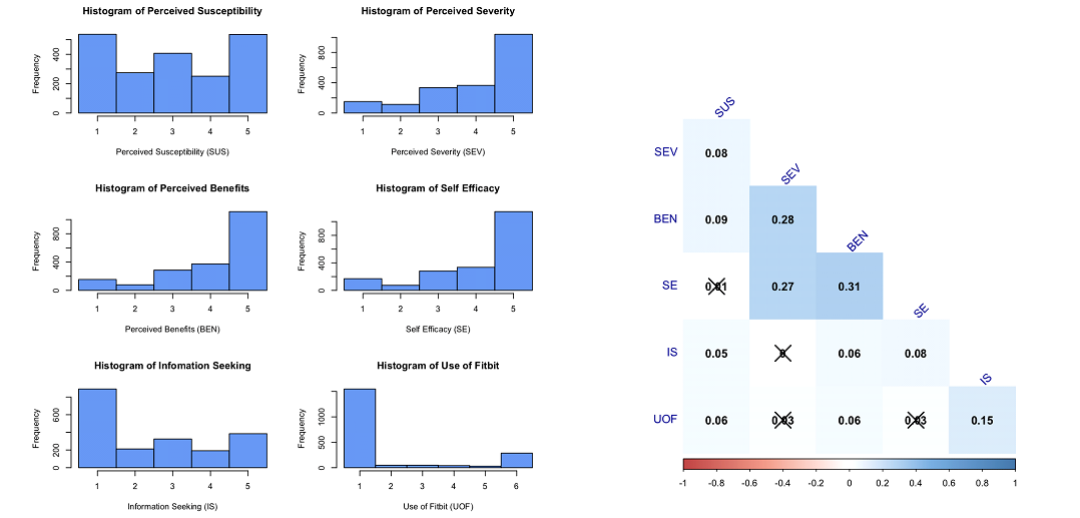
Histograms (left) and a correlogram (right) of measurement variables.

A total of 2006 participants were recruited to participate in the study. Table 2 presents the sample’s demographic characteristics. The majority of BMI scores were >25, indicating obesity (n=1301, 66%). More than half of the participants were female (n=1183, 59%), and males accounted for approximately 41% (n=823) of the sample. Most participants were married (n=1374, 69%), and over half had a college degree (n=1100, 55%) and were White or Caucasian (n=1126, 56%), followed by African Americans, Hispanics/Latinos, others, and Asians. Table 3 and Figure 2 also present summary statistics and histograms of the measurement variables.

**Table 2:** Demographic characteristics (N=2006).

Variable		Participants, n (%)	Perceived susceptibility, mean (SD)	Perceived severity, mean (SD)	Perceived benefits, mean (SD)	Self-efficacy, mean (SD)	Information seeking, mean (SD)
**Gender**							
	Male	823 (41.03)	2.87 (1.54)	3.98 (1.25)	3.99 (1.26)	4.04 (1.28)	2.33 (1.49)
	Female	1183 (58.97)	3.09 (1.54)	4.05 (1.26)	4.19 (1.21)	4.16 (1.25)	2.60 (1.62)
**Age group**							
	<30 years	195 (9.72)	2.54 (1.46)	3.65 (1.17)	4.00 (1.13)	4.10 (1.08)	3.09 (1.39)
	30-50 years	710 (35.39)	3.04 (1.52)	4.06 (1.24)	4.16 (1.18)	4.16 (1.23)	2.95 (1.59)
	>50 years	1101 (54.89)	3.05 (1.57)	4.06 (1.28)	4.09 (1.29)	4.07 (1.32)	2.09 (1.48)
**Ethnicity**							
	White/Caucasian	1126 (56.13)	2.87 (1.50)	4.06 (1.22)	4.09 (1.22)	4.10 (1.28)	2.34 (1.51)
	Hispanic/Latino	189 (9.42)	3.30 (1.56)	4.08 (1.14)	4.26 (1.08)	4.17 (1.14)	3.12 (1.62)
	African American	523 (26.07)	3.20 (1.59)	3.88 (1.37)	4.09 (1.31)	4.08 (1.30)	2.60 (1.63)
	Native American/Pacific Islander	14 (0.7)	3.79 (1.67)	4.86 (0.53)	4.29 (1.27)	4.57 (0.85)	1.82 (1.72)
	Asian	32 (1.6)	3.01 (1.58)	4.03 (1.20)	4.31 (1.06)	4.16 (1.14)	1.97 (1.49)
	Others	122 (6.08)	2.73 (1.58)	3.98 (1.30)	4.04 (1.31)	4.13 (1.23)	2.40 (1.62)
**BMI (kg/m²)**							
	<18.5	41 (2.07)	1.98 (1.39)	4.05 (1.34)	4.15 (1.32)	3.68 (1.67)	2.34 (1.49)
	18.5-25	642 (32.36)	2.46 (1.54)	4.05 (1.25)	4.17 (1.20)	4.10 (1.31)	2.48 (1.55)
	>25	1301 (65.58)	3.28 (1.47)	4.00 (1.26)	4.08 (1.25)	4.13 (1.23)	2.51 (1.59)
**Yearly income ($US)**							
	Low (<$50,000)	1017 (50.70)	3.05 (1.59)	3.95 (1.32)	4.05 (1.30)	3.99 (1.34)	2.44 (1.61)
	Medium ($50,000-$150,000)	811 (40.43)	2.97 (1.50)	4.09 (1.20)	4.19 (1.14)	4.23 (1.18)	2.49 (1.54)
	High (>$150,000)	178 (8.87)	2.84 (1.51)	4.11 (1.13)	4.07 (1.23)	4.26 (1.15)	2.79 (1.49)
**Marital status**							
	Married	1374 (68.50)	3.02 (1.54)	4.08 (1.23)	4.11 (1.25)	4.10 (1.30)	2.83 (1.61)
	Single	632 (31.51)	2.95 (1.56)	3.88 (1.30)	4.12 (1.20)	4.13 (1.20)	2.34 (1.54)
**Education**							
	Low (<high school graduate)	639 (31.85)	3.04 (1.63)	4.05 (1.30)	4.04 (1.33)	3.97 (1.35)	2.21 (1.57)
	Medium (college graduate)	1100 (54.84)	2.97 (1.53)	3.99 (1.25)	4.12 (1.21)	4.15 (1.23)	2.57 (1.57)
	High (master’s degree and above)	267 (13.31)	3.02 (1.44)	4.05 (1.19)	4.24 (1.12)	4.25 (1.18)	2.85 (1.53)

**Table 3:** Summary statistics: mean (SD) and correlation matrix (Spearman correlation coefficients and *P* values).

Variable	Mean (SD)	Perceived susceptibility	Perceived severity	Perceived benefits	Self-efficacy	Information seeking	Use of wearable activity trackers
Perceived susceptibility	2.997 (1.548)	1					
Perceived severity	4.017 (1.258)	0.081 *(P*<*.*001)	1				
Perceived benefits	4.109 (1.235)	0.088 *(P*<.001)	0.275 (*P*<.001)	1			
Self-efficacy	4.107 (1.266)	0.013 *(P*=.56)	0.272 (*P*<.001)	0.309 (*P*<.001)	1		
Information seeking	2.490 (1.576)	0.055 (*P*=.01)	0.000 (*P*=.99)	0.064 (*P*=.004)	0.076 (*P*=.001)	1	
Use of wearable activity trackers	1.909 (1.815)	0.058 (*P*=.01)	0.032 (*P*=.15)	0.055 (*P*=.01)	0.032 (*P*=.15)	0.145 *(P*<.001)	1

### Model Development

The chi-square goodness-of-fit statistic is an index of model adequacy for which a nonsignificant value indicates a good fit of the model to the data. Our work used 4 additional fit indexes to decide how well the specified model explains the data: the comparative fit index (CFI), the root mean square error of approximation (RMSEA), the standardized root mean square residual (SRMR), and the Tucker–Lewis index (TLI). CFI compares the difference between the chi-square value and degrees of freedom (df) of the null (independent) model to the difference between the chi-square value and df of the hypothesized model. Then, this difference is divided by the difference between the chi-square value and df of the null model. CFI is not sensitive to sample size and the recommended cut-off value is ≥0.90 [43]. RMSEA presumes that the best-fitting model has an RMSEA value of 0, that is, an index close to 0 means the model has an excellent fit and a larger index indicates that the model is poor to fit the data. The recommended cut-off value is <0.06 [43]. SRMR measures the average of standardized residuals between the observed covariance and the hypothesized covariance. An SRMR value of 0 represents a perfect fit; a bigger index means that the model is poor to fit the data. The recommended cut-off value is <0.06 [43]. TLI measures the ratio of the difference between the ratio of the chi-square value to the df of the null model and the ratio of the chi-square value to the df of the hypothesized model to the difference between the ratio of the chi-square value to the df of the null model and hypothesized model. The recommended cut-off value is ≥0.95 [43]. After checking the model adequacy, individual paths were tested by the *z* test.

**Figure 3:**
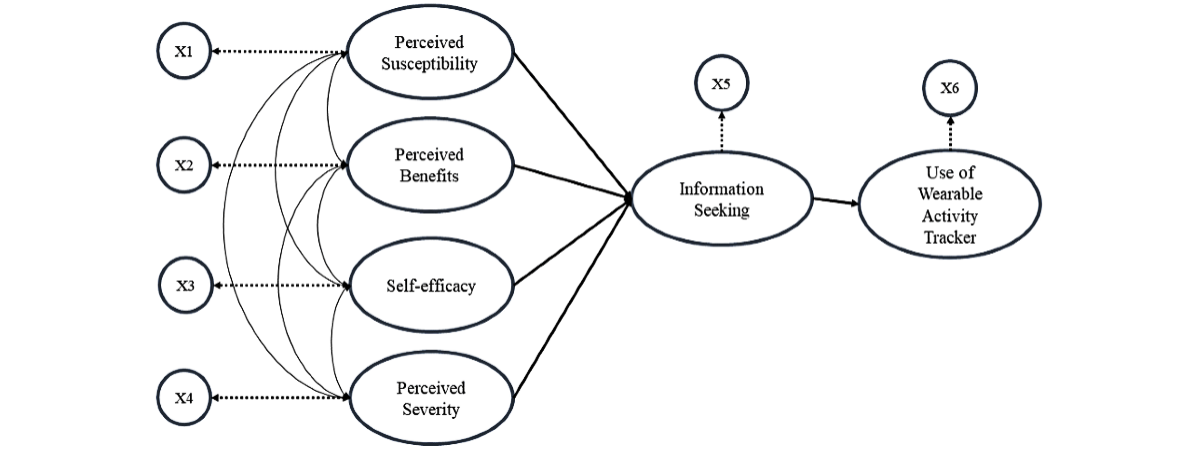
The initial model. X refers to the measurement variable (the mean score of each variable).

The structural model (path model) is a special case of structural equation modeling (SEM). In the structural model, each measurement variable connects to each construct, that is, there exists a one-to-one mapping between constructs and measurement variables, and measurement errors become 0. The initially proposed model 1 is represented in Figure 3. Model 1 posits that perceived susceptibility (SUS), perceived severity (SEV), perceived benefits (BEN), and self-efficacy (SE) together contribute to information seeking (IS), and IS contributes to the use of wearable activity trackers (UOW).

We assumed that all exogenous latent variables were correlated, and all data were analyzed using R, version 3.6.1 (The R Project for Statistical Computing). The *lavaan* package in R is an open-source program that is extremely powerful and flexible for SEM. The factor analytic models and the path model for model 1 were as follows:

- *x*_1_: the measurement variable of SUS;
- *x*_2_: the measurement variable of SEV;
- *x*_3_: the measurement variable of BEN;
- *x*_4_: the measurement variable of SE;
- *x*_5_: the measurement variable of IS;
- *x*_6_: the measurement variable of UOW;
- *ξ*_1_: the exogenous variable (SUS);
- *ξ*_2_: the exogenous variable (SEV);
- *ξ*_3_: the exogenous variable (BEN);
- *ξ*_4_: the exogenous variable (SE);
- *η*_1_: the endogenous variable (IS);
- *η*_2_: the endogenous variable (UOW).

The factor analytic models for the exogenous and endogenous variables were as follows:




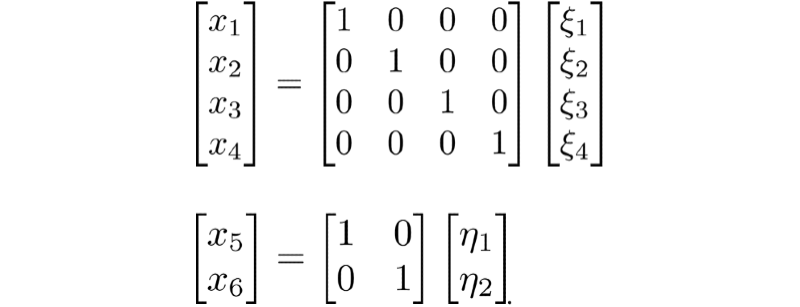




The path model of structural coefficients was as follows:




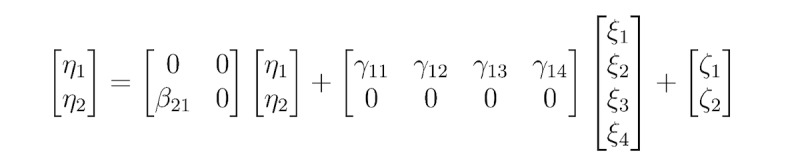




where *β*_21_ is the coefficient of *η*_1_ on *η*_2_, 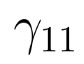 is the coefficient of *ξ*_1_ on *η*_1_, 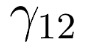 is the coefficient of *ξ*_2_ on *η*_1_, 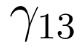 is the coefficient of *ξ*_3_ on *η*_1_, 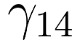 is the coefficient of *ξ*_4_ on *η*_1_, and 
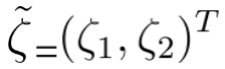
 is the vector of equation errors to predict *η*_1_ and *η*_2_. Since there is a one-to-one mapping between constructs and measurement variables, the measurement errors are zero.

Our model fit statistics included the following: chi-square, CFI, RMSEA, SRMR, and TLI. For the initial model, the SEM analysis indicated that the *P* value of the chi-square test was .07, which is greater than .05, indicating that this structural model fit the data well. The CFI value was 0.991, the RMSEA value was 0.024, the SRMR value was 0.017, and the TLI value was 0.967. Table 4 shows the parameter estimates, standard errors, test statistics (*z* value), and *P* values for each path in model 1. The coefficients (*β*s and 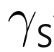) were estimated by maximum likelihood estimation. Susceptibility, benefits, and self-efficacy significantly predicted information seeking, and information seeking was significantly reflected in wearable activity tracker use (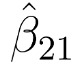
=.137, *P*<.001). However, perceived severity did not give rise to information seeking (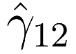
=–0.031, *P*=.30). This may indicate that this model may not be adequate and needs improvement. Therefore, we considered an alternative model.

**Figure 4:**
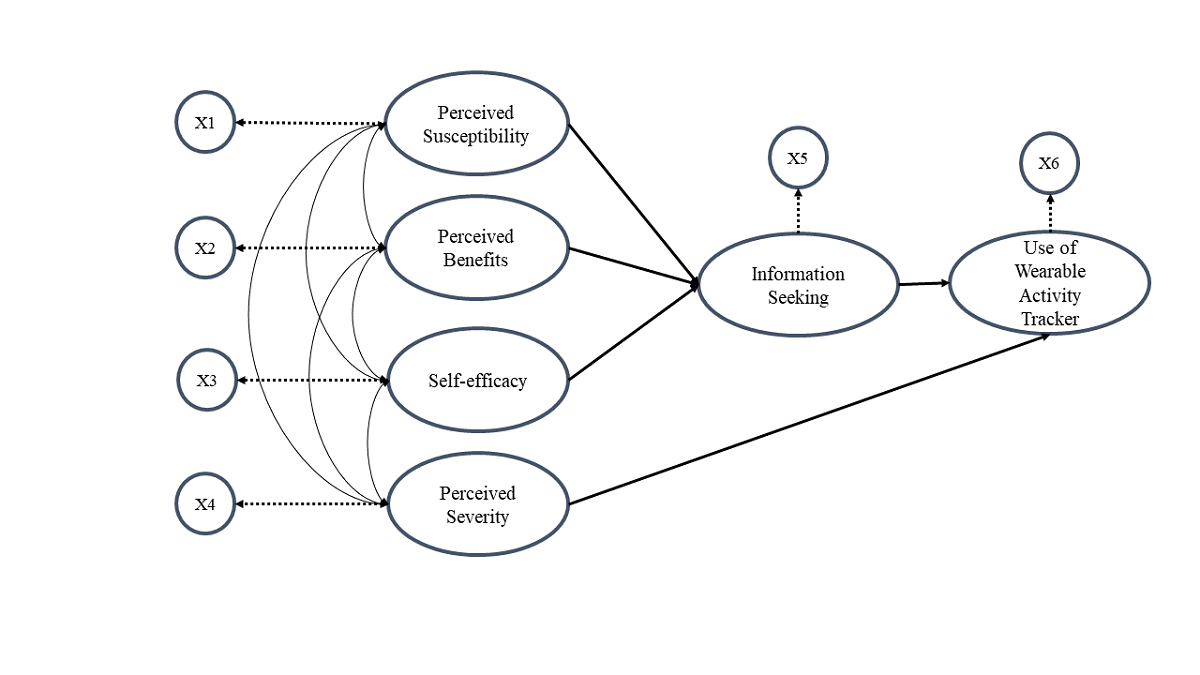
The proposed second model. X refers to the measurement variable (the mean score of each variable).

Our second model posits that susceptibility, benefits, and self-efficacy perceptions together contributed to information seeking, while perceived severity and information seeking together contributed to wearable activity tracker use (Figure 4). The only difference was that severity perceptions directly predicted wearable activity tracker use.

**Table 4:** Parameter estimates for model 1.

Hypothesized relations between constructs	Parameter estimate^a^	SE	*z* value	*P* value
Perceived susceptibility → Information seeking	0.057 (0.056)	0.023	2.516	.01^b^
Perceived severity → Information seeking	­–0.031 (–0.025)	0.030	­–1.044	.30
Perceived benefits → Information seeking	0.077 (0.061)	0.031	2.532	.01^b^
Self-efficacy → Information seeking	0.089 (0.071)	0.030	2.975	.003^c^
Information seeking → Use of wearable activity tracker	0.137 (0.119)	0.026	5.373	<.001^c^

^a^The values in parentheses are standardized estimates.

^b^Significance at the .05 level.

^c^Significance at the .01 level.

The factor analytic models for exogenous and endogenous variables were the same as model 1, but the path model of the structural coefficients was different from the model 1. The path model of structural coefficients was as follows:




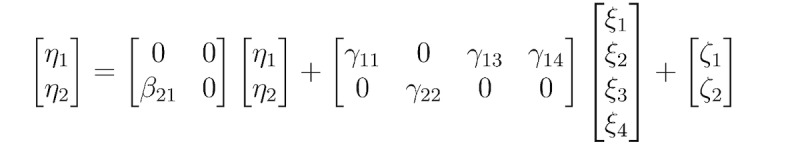




where *β*_21_ is the coefficient of *η*_1_ on *η*_2_, 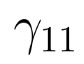 is the coefficient of *ξ*_1_ on *η*_1_, 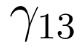 is the coefficient of *ξ*_3_ on *η*_1_, 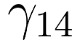 is the coefficient of *ξ*_4_ on *η*_1_, and 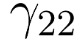 is the coefficient of *ξ*_2_ on *η*_2_. Note that the only difference between model 1 and model 2 is the matrices in the middle term.

Table 5 shows model fit statistics and confirms that the second model has a better fit than the initial model since CFI and TLI were higher, and chi-square, RMSEA, and SRMR were lower. As the second model supports all hypotheses (Table 4) with better fit indicator scores, we choose the second model as the final model.

Table 6 demonstrates parameter estimates for our final model (ie, model 2). In short, susceptibility, benefits, and self-efficacy perceptions significantly predicted information seeking, which, in turn, indirectly predicted wearable activity tracker use. Compared to the initial model, severity perceptions directly predicted wearable activity tracker use (Table 6).

**Table 5:** Model fit statistics for model 2.

Model	Chi-square	*P* value	Model fit^a^			
			CFI^b^	RMSEA^c^	SRMR^d^	TLI^e^
1	8.665	.07	0.991	0.024	0.017	0.967
2	5.811	.21	0.997	0.015	0.012	0.987

^a^Cut-off for good fit: CFI≥0.90, RMSEA<0.06, SRMR<0.06, and TLI≥0.95.

^b^CFI: comparative fit index.

^c^RMSEA: root mean square error of approximation.

^d^SRMR: standardized root mean square residual.

^e^TLI: Tucker–Lewis index.

**Table 6:** Parameter estimates for model 2.

Hypothesized relations between constructs	Parameter estimate^a^	SE	*z* value	*P* value
Perceived susceptibility → Information seeking (hypothesis 1)	0.056 (0.055)	0.023	2.455	.01^b^
Perceived benefits → Information seeking (hypothesis 2)	0.071 (0.056)	0.030	2.372	.02^b^
Self-efficacy → Information seeking (hypothesis 3)	0.082 (0.066)	0.029	2.822	.005^c^
Perceived severity → Use of wearable activity trackers (hypothesis 4)	0.063 (0.044)	0.032	1.986	.047^b^
Information seeking → Use of wearable activity trackers (hypothesis 5)	0.136 (0.118)	0.026	5.343	<.001^c^

^a^The values in parentheses are standardized estimates.

^b^Significance at the .05 level.

^c^Significance at the .01 level.

**Figure 5:**
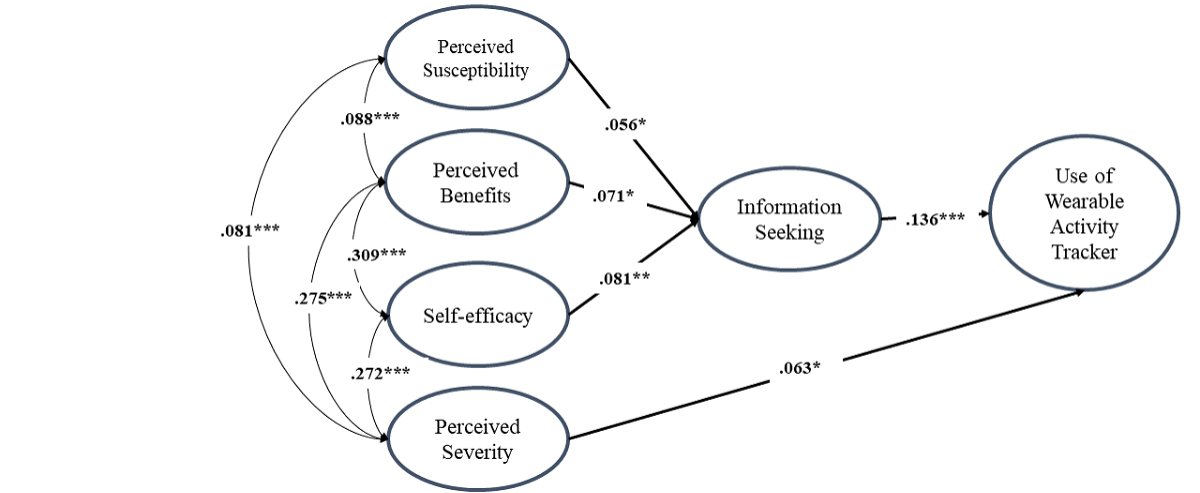
Results of the final Integrated Model of Wearable Activity Tracker use. Asterisks indicate the level of significance for each path's *P* value.

Since the distributions of variables appeared to be skewed, we also considered the asymptotic distribution-free (ADF) estimator. Even though it is well known that the maximum likelihood estimator is relatively robust to violations of normality assumptions, and a large sample reduces the problem of multivariate nonnormality, it is worth checking our models with ADF. Consistent with maximum likelihood estimation, ADF estimates (not shown) had very similar values to the maximum likelihood estimates, and their *P* values were also very close to the *P* values of the maximum likelihood estimates. In the initial model, ADF indicated that SEV (perceived severity) was not significant, whereas all paths were significant in the second model (see Figure 5 for our final model).

## Discussion

### Principal Results

Our multiple analyses generated a meaningful model of health care technology use that provides several contributions to theory and practice.

First, it supports the application of the HBM in the use of wearable activity trackers, which was not present in most existing works. The key factors were identified within two paths: perceived benefits, perceived susceptibility, and self-efficacy influence health information–seeking behaviors, while perceived severity is directly related to adults’ use of wearable fitness trackers. It is meaningful to see how those factors are influencing the actual application of wearable activity-tracking devices with a varying range in significance and directional relationships.

According to standardized estimates, we may identify a relatively stronger predictor of health information–seeking behaviors: self-efficacy. This finding was consistent with a previous study [22] that found self-efficacy to be the strongest predictor of health-related behaviors. In turn, the linkage between health information–seeking and wearable health technology usage showed the largest impact. With such highlighted findings, this study validates the succinct and powerful model that re-evaluates and reorganizes previous works.

Interestingly, obesity severity perception was not related to online health information seeking, but rather directly related to adults’ use of wearable fitness trackers. One possible explanation for this could be the nature of the predictor. Perceived severity has strong direct influences on behavioral outcomes, as supported by Lee and Kim [25]. Carpenter [20] also confirmed such discrepancy can happen, presumably due to its small effect size. Specifically, 18 studies with 2702 subjects were used to determine whether measures of the 4 variables (eg, perceived barriers, perceived benefits, perceived susceptibility, perceived severity) could universally predict health behaviors, regardless of the context (prevention vs treatment behaviors). It was found that prevention and treatment behaviors moderated each of the 4 HBM variables’ predictive power; for prevention behaviors in particular, perceived severity (*r*=0.14) was the second strongest predictor, followed by perceived barriers (*r*=0.22). In short, the above findings address two concerns: (1) additional research is needed to explore the predictive power of severity perceptions on other health-related contexts, as suggested by our data, and (2) future research should avoid the continued use of the direct and universal effects version of the HBM, as illustrated by our results.

Another thing to note is that our integrated model was confirmed by a large national cohort comprising over 2000 people, and the context was tailored to use of a specific technology—wearable activity trackers. Previously, the TAM has been the only theoretical framework that dominantly explained the actual use of new technology. Our study contributes to the discipline by providing a new theoretical model combining the health beliefs and information-seeking behaviors for wearable activity tracker use. This could lead to additional contributions, such as gaining evidence-based knowledge on the precedents of wearable fitness trackers usage to promote physical activities and improve the outcomes of obesity interventions, and further evaluating its long-term effects. Using the proposed IMWAT, the mechanism underlying wearable health technology use can be explained.

### Limitations and Future Works

This study has limitations that should be addressed in future studies. First, we noticed that our endogenous variable, wearable activity tracker use, was skewed. This may have hindered the process of normal SEM; hence, we ran the same analysis to assess model fit and whether the paths were significant using the ADF estimator, which does not require any assumption in the data distribution. Consequently, the analysis with ADF generated the same result as our model tested with maximum likelihood, which does not make this limitation a major issue.

Considering that our sample was skewed to those who were obese (34.4% were healthy adults, while 65.6% reported BMI scores >25), a stratification analysis by individuals’ weight status still needs to be explored in future research. To promote the wider use of wearable activity trackers, future studies also need to examine what triggers adults to adopt wearable health technologies and motivates them to continue using these devices.

Finally, future studies could apply more variables from other major theories of health behaviors, such as the Theory of Planned Behavior. For instance, perceived behavioral control could be a meaningful addition to the current model. This variable is closely related to self-efficacy in the HBM, since they both reflect people’s confidence in performing health behaviors [44].

### Practical Implications and Conclusions

This study provides a theory-driven mathematical model of how different interactions between individual beliefs and multifactors influence wearable fitness tracker use among both obese and healthy adults. Our model holds several managerial implications for health informatics and health care practitioners utilizing wearable activity trackers for public obesity intervention programs [45]. Changing individuals’ daily activity is not an easy task, but practitioners should focus on effective communication strategies that make users feel that use of wearable activity trackers is not a barrier to overcome but a beneficial way of managing oneself. Similarly, health informatics and health care practitioners could benefit from promoting the significance and severity of obesity to their target health consumers, which, in turn, can lead to their actual uptake of health technology (ie, behavior change), as suggested by our model.

As Feng and colleagues [45] pointed out, not much research attention has been paid to healthy populations, who, despite being generally healthy, tend to have distinct personal health information management needs. With the rising popularity of wearable fitness trackers such as Fitbit [13], it is now extremely important to offer such wearable technologies as a complement to traditional health care services, rather than as a substitute, because of accuracy and validity of the data they provide [14].

All in all, the state-level sample of over 2000 people produced nationally applicable results, ensuring the generalization of this study to a wider population and supporting the practical use of the results. The variables employed in this model will assist wearable health technology product developers and designers.

## Abbreviations

ADF estimator: asymptotic distribution-free estimator
BEN: perceived benefit
CFI: comparative fit index
df: degrees of freedom
HBM: Health Belief Model
IMWAT: Integrated Model of Wearable Activity Tracker
IS: information seeking
RMSEA: root mean square error of approximation
RQ: research question
SE: self-efficacy
SEV: perceived severity
SRMR: standardized root mean square residual
SUS: perceived susceptibility
TAM: Technology Acceptance Model
TLI: Tucker–Lewis index
UOW: use of wearable activity trackers
